# Editorial: Tissue regeneration using dental stem cells

**DOI:** 10.3389/fcell.2024.1401476

**Published:** 2024-03-22

**Authors:** Marco Tatullo, Ian Ellis, Mohammad Islam

**Affiliations:** ^1^ Department of Translational Biomedicine and Neuroscience (DIBRAIN), University of Bari Aldo Moro, Bari, Italy; ^2^ School of Dentistry, University of Dundee, Dundee, United Kingdom; ^3^ MIRROR—Medical Institute for Regeneration and Repairing and Organ Replacement, Interdepartmental Center, University of Bari Aldo Moro, Bari, Italy

**Keywords:** tissue regeneration, dental stem cells (DSCs), biomaterial, growth factors, bone regenaration, immunomodulation

Tissue regeneration is a multidisciplinary field that combines various aspects of biology, medicine, materials science, and engineering. The three main pillars of tissue engineering are: biomimetic biomaterials, growth factors able to drive cell fate, and, most importantly, stem cells. The complex interaction between resident stem cells and the local environment is a multifactorial process, regulated by multiple pathways able to actively influence a wide range of biological processes, from cell division to multilineage differentiation (Santilli et al.).

In this landscape, several studies have undoubtedly demonstrated that dental and periodontal tissues contain a significant amount of highly immature mesenchymal stem cells (MSCs). Dental MSCs (DMSCs) have been found in various dental tissues; moreover, DMSCs have gained growing interest from the scientific community because of their easy accessibility, and thanks to their high strategic plasticity both in dental tissues repair and in regenerating several types of human tissues, including bone, cartilage, muscle, and neural tissue. Despite oral tissues not being the most used niches of MSCs, we should carefully rethink the role of DMSCs in tissue regeneration, as their use may lead to a significant advance of knowledge in regenerative dentistry, impacting on the future treatments of various systemic diseases (Namjoynik et al., 2023; Tatullo et al., 2023).

This Research Topic has investigated a wide range of Research Topic, confirming the valuable potentialities of DMSCs in tissue regeneration. Several approaches have been proposed to optimize and enhance the regenerative potential of MSC, such as the use of media conditioned with different growth factors, or cytokines and bioactive molecules (Liu et al., 2022; Ueda et al., 2022). However, these biological strategies alone were deemed not to have reached their potential in some specific clinical conditions (Chouaib et al., 2022); thus, the researchers have added physical factors to improve cell proliferation. Using this premise, studies on various light sources, including LASERs and light-emitting diode (LED), were reviewed, and indicated that photo-biomodulation could be a good aid in those regenerative protocols aimed at stimulating periodontal ligament stem cells (PDLSCs) to repair damaged dental tissues. However, the review revealed that there were a lot of ambiguity in these protocols and could have a conflict of interests as they were funded by the LASER companies (Ponnaiyan et al.).

Another important Research Topic discussed here is the importance of bioactive scaffolds, as they can create an optimal microenvironment for MSCs. In fact, biomaterials, such as polymers, hydrogels, or composites, are well known and safely used in the production of scaffolds to be characterized by 3D superficial morphology that should emphasize the concept of “sensing surface.” This concept, for example, may involve the presence of a superficial presence of porosity with specific size that promote the MSCs adhesion, growth, and differentiation. Moreover, biomaterials need to contain specific molecules or factors that mimic the target tissue microenvironments, to promote a targeted tissue regeneration (Gaharwar et al., 2020).

Recently, biomaterials from foods or plants have been studied. Due to its mechanical properties, absence of any cell toxicity, and capacity to create three-dimensional structures, chitin provides an optimal environment for promoting stem cell viability, proliferation, and differentiation (Duminis et al., 2023). For the first time, Zawadzka-Knefel et al. investigated the biological responses of human dental pulp stem cells (DPSCs) on purified 3D chitin-based scaffolds: these scaffolds were obtained from the marine demosponge *Aplysina fistularis* and were then modified using hydroxyapatite (HAp). The authors demonstrated that the chitin-based scaffolds, especially those modified with HAp, have the potential of improve DPSCs adhesion and differentiation towards osteoblasts and odontoblasts.

The bone morphogenetic proteins (BMPs) are recognised as among the most important players in many processes related to the formation and maintenance of various tissues, including bone, cartilage, and muscle (Akiyama et al., 2023). In addition, BMPs hold significant potential as bioactive factors for tooth root regeneration for regulating signalling pathways in various stages of tooth root development. In particular, BMPs promote the formation of Hertwig’s epithelial root sheath (HERS) and the differentiation of DMSCs into odontoblasts, cementoblasts, and osteocytes, contributing to the formation of dentin and periodontal tissues (Liu et al.).

While BMPs play an important role in cell crosstalk, the role of plasma membrane lipids is particularly important in cell division, as they can influence the multilineage differentiation ability. The small, heterogeneous, and highly dynamic lipid domains in the cell membranes, called *lipid rafts*, are involved in a variety of cellular processes, including receptor-mediated signal transduction, and they can effectively regulate cellular signalling, including proliferation, differentiation, apoptosis, autophagy, necrosis, stress responses, inflammation, and senescence (Santilli et al.). Moreover, recent evidence has also shown that lipid rafts are involved in the survival, death, and metastasis of cancer cells, making them a promising target for cancer therapy (Li et al., 2022).

It is well known that controlling and eliminating inflammation, and modulating immunity, may increase tissue regeneration. As widely reported in the literature, DMSCs can exhibit anti-inflammatory and immunomodulatory potential, making them promising candidates for treating many inflammatory and autoimmune diseases (Li et al.). As happens with other stem cells, the immunomodulatory potential of DMSCs is regulated by the release of molecules and factors in the extracellular environment through specific carriers, such as extracellular vesicles (EVs) or exosomes (EXOs). Interestingly, EVs may play a pivotal role in stem cell-based therapies (Codispoti et al., 2018), even if, the release of EVs, is susceptible to the lipid rafts’ perturbations (Feng et al., 2020). In this context, Tian et al. (2023) have demonstrated that EVs derived from hypoxic pre-conditioned DPSCs seems to ameliorate inflammatory-related osteolysis, through an intense modulation of macrophage polarisation, and an inhibition of osteoclastogenesis obtained by targeting Nuclear Factor Kappa B Subunit 1 (NF-kB1) which plays a crucial role in the inflammatory response and osteoclast formation.

In conclusion, these studies have essentially confirmed the *in vitro* close interaction between DMSCs and several factors that have different roles in tissue regeneration. It would also be strategic to investigate the regulatory mechanisms that are able to influence DMSCs behaviour, for a better safety profile in their future applications on patients. In fact, although DMSCs have been shown to have important pros, undoubtedly, important Research Topic such as safety remain. Thus, the future studies should focus on their safe and predictable use in daily surgical therapies, firstly in dental regeneration, and then in systemic degenerative diseases.
